# Seasonal and gender-specific patterns in prescriptions for hypnotic and sedative medications in primary care

**DOI:** 10.3389/fpsyt.2026.1785512

**Published:** 2026-05-20

**Authors:** Małgorzata Zofia Romaszko, Katarzyna Glińska-Lewczuk, Leszek Gromadziński, Julia Gajkowska, Michal Majewski

**Affiliations:** 1 Department of Pharmacology and Toxicology, School of Medicine, Collegium Medicum, University of Warmia and Mazury, Olsztyn, Poland; 2 Department of Water Management and Climatology, University of Warmia and Mazury in Olsztyn, Olsztyn, Poland; 3 Department of Cardiology and Internal Medicine, University of Warmia and Mazury in Olsztyn, Olsztyn, Poland; 4 Department of Cardiology and Internal Diseases, Military Institute of Medicine, Warsaw, Poland

**Keywords:** circadian rhythm, daylight saving time, hypnotics, primary care, seasonality, sedatives

## Abstract

**Introduction:**

Population-level prevalence of sleep disorders can be assessed using prescription data for hypnotic and sedative medications. Such prescribing patterns exhibit seasonality that may be linked to variations in daylight exposure. The aim of this study was to analyze temporal trends in prescriptions for drugs with sedative-hypnotic properties.

**Methods:**

Prescription data for hypnotics and sedatives were analyzed retrospectively and stratified by month, year, and patient gender. Seasonal patterns, associations with day length, and the effects of transitions between daylight saving time and standard time were examined. Changes in prescription numbers during the COVID-19 pandemic were also assessed. Relative differences in prescription counts were evaluated using incidence rate ratios (IRR).

**Results:**

Prescription numbers were lowest in summer (May–August) and highest in winter and early spring. Increasing day length was significantly associated with reduced prescription rates. A decline in prescriptions occurred earlier and was more pronounced in men (February–September; IRR 0.88–0.95), whereas in women the changes were weaker and mainly limited to summer months (June–August; IRR 0.94–0.97), with a slight increase observed in February. During the COVID-19 pandemic, prescription numbers decreased significantly. Transitions between standard and daylight saving time exerted measurable short-term effects on sleep-related health at the population level.

**Conclusions:**

Based on data from a single primary care center in Poland, prescribing patterns for hypnotic and sedative medications demonstrate clear seasonality and significant gender differences. Longer daylight exposure and transitions to daylight saving time are associated with lower prescription rates. The COVID-19 pandemic substantially disrupted previous trends in sleep medication prescribing, which may be related to reduced access to healthcare services and changes in healthcare delivery. In addition, transitions between standard and daylight saving time were associated with statistically significant short-term changes in prescription rates.

## Introduction

Sleep is fundamental for the body’s regenerative processes, hence sleep disorders are considered a significant public health issue (1). The most common sleep disorder reported to general practitioners (GPs) is insomnia, understood as difficulty falling asleep, staying asleep, or waking up too early – despite conditions conducive to sleep – and insufficient sleep, most often caused by lifestyle factors (shift work, exposure to screens) (2–4). What generated our interest was the presence (or absence) of population-level seasonality in sleep disorders. The data available in the literature provide ambiguous answers. Mattingly SM et al. described seasonal effects regarding wake-up time and sleep duration: waking up occurs earlier and the length of sleep is reduced in spring as compared to the winter season (5). We believe that an attempt should be made to assess the range of the phenomenon using an indirect method – by analyzing the consumption of potentially hypnotic drugs. Prescription data may serve as a proxy for population-level patterns and can be used as a supportive tool in the analysis of sleep disorders; however, they reflect prescribing behavior rather than the true prevalence of these conditions. Such studies are, however, scarce. In the only report that we were able to identify, from Croatia, Gradiški IP et al. noted monthly differences in the use of drugs such as zolpidem, nitrazepam, flurazepam, and midazolam, but found no evident seasonality (6).

In Scandinavian countries, where differences in the length of the day depending on the season constitute a kind of “natural experiment, “ Titova et al. suggested the existence of seasonal sleep disorders. They revealed that patients sleep less during the summer months, when exposure to sunlight is particularly intense (7). Similar results were presented by Svirtsen B et al. based on data from northern regions of Norway, which documented worse sleep quality during the winter months (8).

The COVID-19 pandemictemporarily restricted access to healthcare services in most European countries (9). The introduction of teleconsultations as the primary form of contact with non-urgent patients without immediate life-threatening conditions significantly limited the possibilities for thorough diagnosis of sleep disorders (10). Hence, this period can be considered a kind of natural epidemiological experiment in which the seasonality of prescriptions for sleep medication was largely determined by patients’ initiative and expectations.

The analysis of seasonal sleep disorders is linked not only to the length of the day/season, factors beyond our control, but also to potentially modifiable events such as transitions from daylight saving time (summer time) to standard time (winter time). In the European Union, the transition from standard time to daylight saving time takes place on the last Sunday in March, while the one from daylight saving time to standard time occurs on the last Sunday in October. This is a recurring, annual event.

The American Academy of Sleep Medicine (AASM) indicates that standard time (winter time) is more in line with the human circadian rhythm, and its consistent use could bring measurable benefits. The transition from standard to daylight saving time contributes to sleep and circadian rhythm disruption (11, 12). Circadian desynchronization can result in insomnia and a reduced subjective and objective sleep quality (13, 14). In 2014 Chunla He et all. published a meta-analysis that revealed a statistically significant increase in the risk of breast cancer (BrCA) among flight attendants, who are chronically exposed to circadian disruption due to irregular time zone changes (15). Significant correlations were reported between circadian rhythm disruption and sleep deprivation and an increased risk of developing conditions such as Alzheimer’s disease, cardiovascular disease, hypertension, and depression (16–18).

Guided by the presented theoretical premises concerning seasonal disturbances in sleep quality, we decided to verify this hypothesis. The simplest and, in our opinion, the most reliable method seemed to be the analysis of the number of prescriptions for drugs with hypnotic properties issued by a primary care physician.

## Methods

In this single-center retrospective analysis, covering 716 242 consultations provided to adults at one of the primary healthcare centers (Pantamed Ltd) in Olsztyn, Poland, which delivers care to approximately 13 000 people (the number of registered patients varies by about 100–200 people each month) and employs several primary care physicians who provide approximately 60 000 medical consultations per year, the number of prescriptions for sleep medication was examined. The documentation of formally adult patients was eligible for the study (≥18 years of age).

In Poland, and particularly in the healthcare center under analysis, electronic prescriptions for medications (the so-called e-prescriptions) have been used since January 2012. Paper prescriptions account for less than 1 per mille of all prescriptions and are issued almost exclusively in emergency situations (software failure, power outage, etc.). The data for the study were obtained with custom software that enables the use of the ATC (Anatomical Therapeutic Chemical) classification, which allows trade names of drugs to be linked to their active agent (Active Pharmaceutical Ingredient, API), (**Table 1**). The software linked patient data with the date of prescription, reason for consultation, and the ATC group (19). This method enabled us to circumvent the problem of drug trade names. The obtained data were divided into three periods: before (1^st^ January 2012 – 31^st^ May 2020), during (1^st^ June 2020 – 30^th^ June 2022) and after the COVID-19 pandemic (1^st^ July 2022 – 31^st^ December 2024). On May 16, 2022, the Polish government issued a revocation of the state of epidemic emergency (20). However, in order to ensure data standardization and due to delayed effects, the analysis period concluded at the end of the following month, and the actual end of the pandemic was determined based on a discriminant analysis (biplot), which allowed for the correct classification of the collected information in 93.9% of cases. Linear Discriminant Analysis (LDA) was used. In addition, we analyzed the number of prescriptions for medications used to treat sleep disorders in the two-week intervals preceding and following the annual seasonal clock change.

**Table 1:** ATC codes included in the analysis.

ATC code	Group name/API (example international trade name)
N05CF	hypnosedatives - cyclopyrrolones/zaleplon, zolpidem and zopiclone (Imovane)
N05CD08	benzodiazepines/midazolam (Versed)
N05CD07	benzodiazepines/temazepam (Restoril)
N05CD06	benzodiazepines/lormetazepam (Noctamid)
N05CD04	benzodiazepines/estazolam (Prosom)
N05CD02	benzodiazepines/nitrazepam (Mogadon)
N05BA12	benzodiazepines/alprazolam (Xanax)
N05BA08	benzodiazepines/bromazepam (Lexotan)
N05BA06	benzodiazepines/lorazepam (Ativan)
N05BA04	benzodiazepines/oxazepam (Serax)
N05BA01	benzodiazepines/diazepam (Valium)
N03AE01	benzodiazepines/clonazepam (Rivotril)

### Statistical analyses

We investigated whether the calendar month was associated with the daily number of prescriptions issued on working days between 1^st^ January 2012 and 31^st^ December 2024. From the total cohort, encompassing each day in the study period (N = 4 749 days), we retained only days on which the primary healthcare clinic was staffed (“On-duty”, N = 3 279). A daily count of prescriptions was the response variable (integer, 0 – 60), while month (1 = January … 12 = December) was treated as a categorical (explanatory) factor.

To reduce the influence of extreme high-frequency users on the results of the analysis of seasonality, in the next step, people (n=140) who had been issued more than 259 prescriptions (mean+2xSD = 259) were excluded from the calculations.

Daily prescription counts constitute non-negative integer data, thus the Poisson generalized linear model (GLM) with log link was chosen as the primary analysis (21). In the present data the Pearson dispersion ratios are close to 1 (1.05 for total prescriptions, 1.04 for males, 1.03 for females), indicating that the Poisson mean–variance equality holds to a satisfactory degree. The Poisson GLM offers the correct distributional basis for our daily count data, yields clinically meaningful IRRs, and passes all goodness-of-fit diagnostics. Its use in pharmacoepidemiology and service research is endorsed by standard biostatistical texts and satisfies STROBE and SAMPL reporting guidelines (22–24).

The factor “month” (12 levels) was entered as a categorical predictor, and the model fit was assessed with a likelihood-ratio χ² test (LRT) comparing the full model with a null model containing only the intercept with January as reference. Goodness-of-fit was assessed with the Pearson χ²/df ratio (called “Pearson dispersion”).

Exponents of the regression coefficients were reported as incidence-rate ratios (IRR) with Wald 95% confidence intervals (CI) (25). All analyses were performed in Python 3.10 with *statsmodels* 0.14 (26).

The effect of the daylight saving time change was assessed by comparing two-week periods before (−14 days) and after (+14 days) time change, separately for women and men, for all calendar days and for working days only. The variables were not normally distributed. For this reason, parametric tests based on the assumption of normality were considered inappropriate. Differences between the two periods were therefore evaluated using the Mann–Whitney *U* test. All tests were two-sided and a *p* < 0.05 was considered statistically significant.

## Results

During the study period (2012–2024), the mean (± SD) number of prescriptions issued per on-duty day was 14.73 ± 5.04. Prescriptions issued to women accounted for 69% of all prescriptions, while those issued to men represented 31%. This gender proportion remained stable throughout the year.

**Figure 1:**
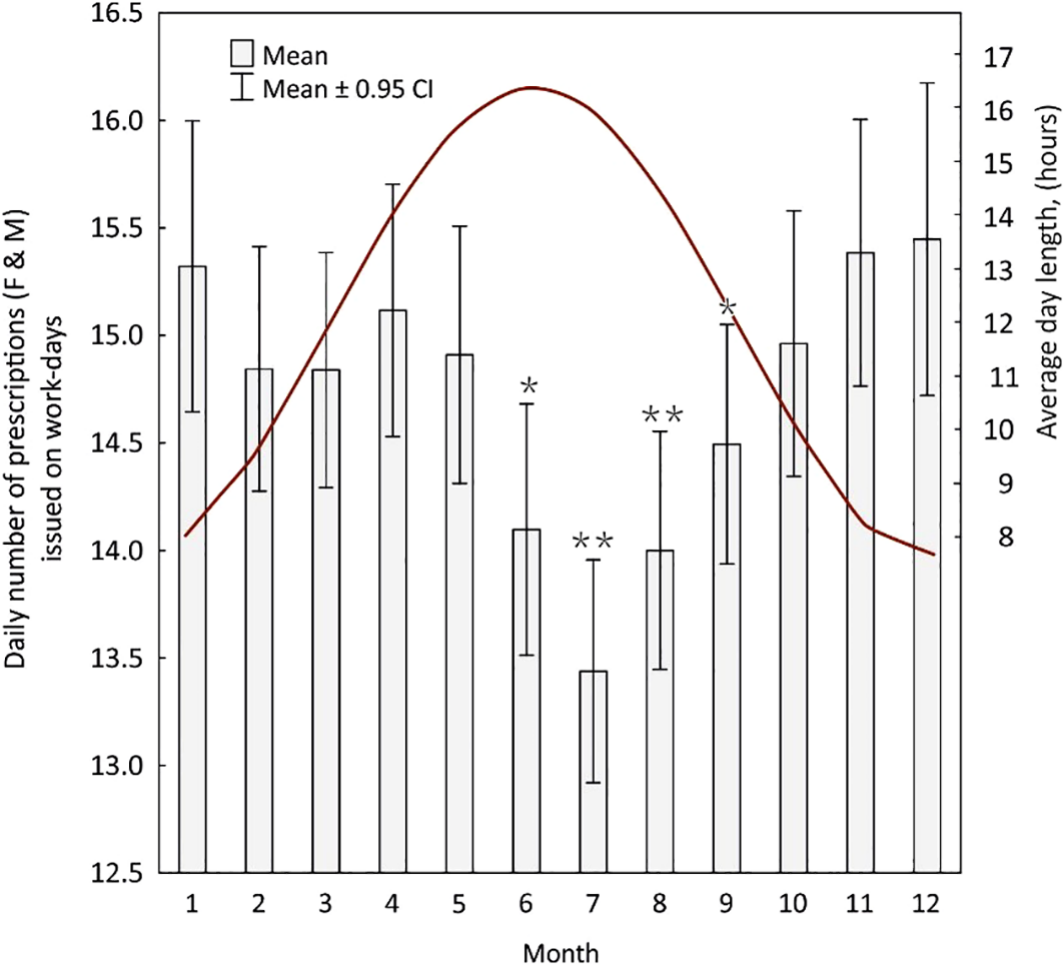
Monthly variation in the mean daily number of prescriptions (<259) issued on workdays (2012–2024). Column-and-whisker plot summarizing 3 279 workdays. The Poisson generalized-linear model with January as the reference month identified periods with significantly lower prescription volumes: June and September (*p* < 0.01; *) and July and August (*p* < 0.001; **). The red line indicates the mean astronomical daylength (hours) through the year, illustrating that reduced summer prescribing coincides with maximal daylight.

Nevertheless, distinct seasonal fluctuations in the total number of prescriptions were revealed in both female and male groups. Prescription activity was highest during winter and early spring and reached its lowest levels in June–August, followed by a gradual increase toward autumn and winter (**Figure 1**).

The likelihood-ratio χ² test, comparing the model including *month* with the null (intercept-only) model, demonstrated a highly significant effect of the month on prescription counts (χ^2^_(11)_ = 62.23, *p* < 0.001). The negative-binomial model yielded consistent results.

These results indicate a robust and reproducible seasonal pattern in prescribing, with significantly lower prescription volumes during months of maximum daylight.

**Table 2** presents the incidence rate ratios (IRR) for the effect of month on daily prescription counts. The overall number of prescriptions declined progressively throughout early summer, reaching its minimum in July (IRR = 0.93; 95% CI: 0.89–0.97), corresponding to approximately 7% fewer prescriptions per day compared with January. The lowest rates occurred consistently from May to August (**Table 3**).

**Table 2:** Incidence rate ratios (IRR) from the Poisson regression model assessing the effect of calendar month on daily prescription counts (reference = January).

Month	IRR	95% CI	*p*
Intercept	10.308	9.999 – 10.626	0.000
February	1.029	0.985 – 1.075	0.193
March	1.022	0.958 – 1.045	0.310
April	1.000	0.980 – 1.067	0.982
May	0.955	0.914 – 0.997	0.038*
June	0.940	0.899 – 0.982	0.006**
July	0.932	0.892 – 0.974	0.002**
August	0.941	0.900 – 0.983	0.006**
September	1.000	0.958 – 1.044	0. 999
October	1.041	0.998 – 1.086	0.065
November	0.995	0.953– 1.039	0.831
December	0.989	0.948 – 1.033	0.628

The table presents monthly IRR values with 95% confidence intervals (CI) and p-values for prescriptions issued on workdays during the period 2012–2024. An IRR < 1.0 indicates a lower prescription rate compared with January, while an IRR > 1.0 indicates a higher rate.Bold values indicate statistically significant results (p < 0.05).

**Table 3:** Results for patients who were prescribed fewer than 259 hypnotic and sedative medications prescriptions (Poisson GLM, working days 2012–2024).

Outcome	Likelihood-ratio test χ² (df = 11)	Significant months (*p* < 0.05)†	IRR range
All patients < 259	62.231	**Jun**, **Jul**, **Aug**, May	0.932 – 0.955
Females < 259 prescriptions	41.692	**Jun, Jul, Aug**	0.910 – 0.945
Males < 259 prescriptions	34.866	**Jun**, **Jul**, **Aug**, Sep	0.905 – 0.950

† bolded months have *p* < 0.01; others 0.01 ≤ *p* < 0.05.

When analyzed by gender, the seasonal pattern was evident in both groups but differed in magnitude and duration. In men, the decline began as early as February and reached minimal values in July (IRR = 0.88; 95% CI: 0.81–0.95). In women, the reduction was narrower, limited to June–August, with the lowest point in August (IRR = 0.94; 95% CI: 0.90–1.00). Notably, women demonstrated a modest but significant increase in February relative to January (IRR = 1.06; *p* = 0.038).

**Figure 2:**
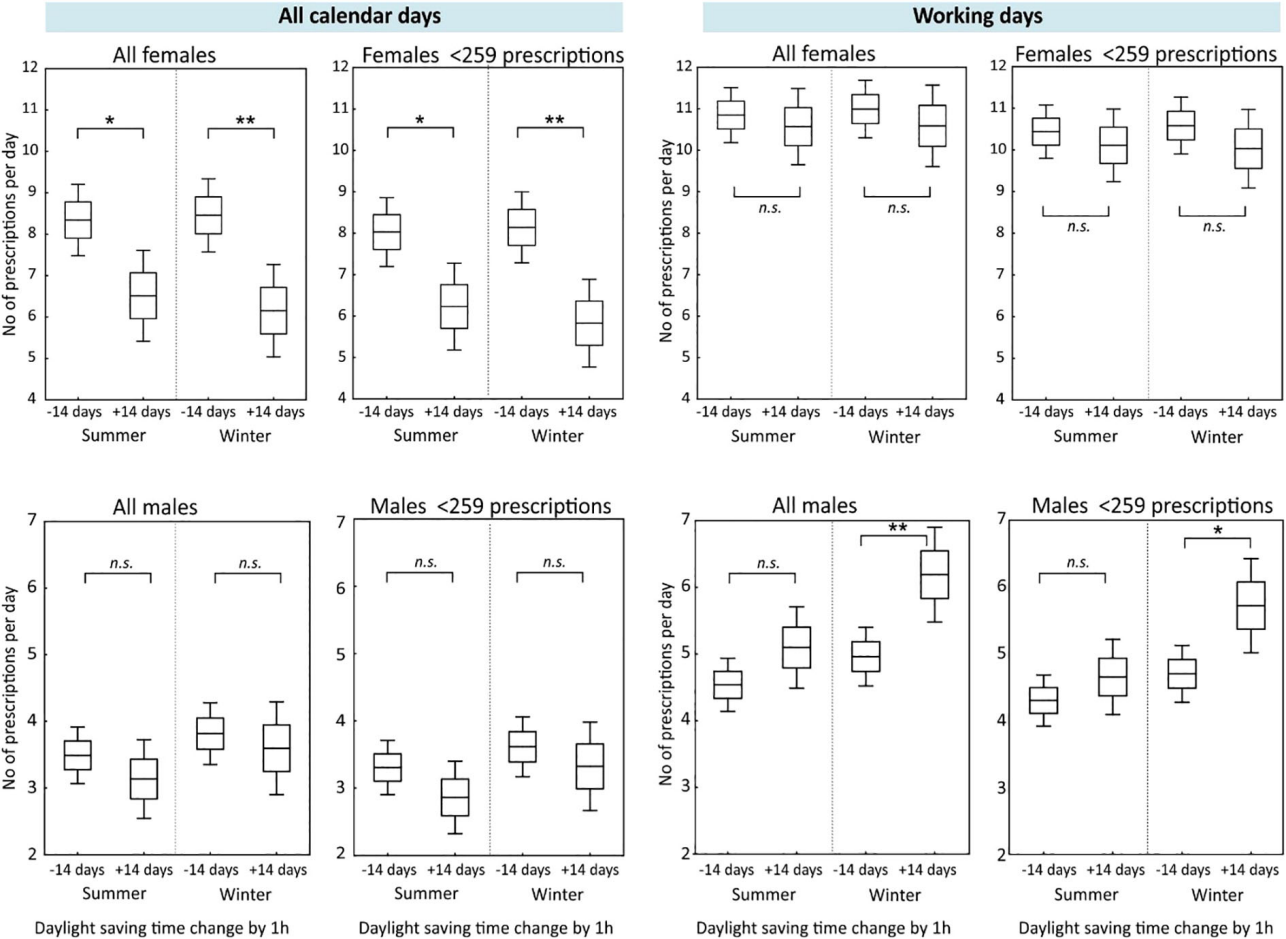
Changes in daily prescription counts 14 days before (−14 days) and after (+14 days) daylight saving time transitions in women and men, as well as in subgroups with fewer than 259 prescriptions during the study period (2012–2024), analysed separately for all calendar days and for working days only. In each panel, the horizontal line represents the mean value, the box indicates the mean ± standard error of the mean (SEM), and the whiskers denote the 95% confidence interval. All calendar days include weekends and public holidays. Daylight saving time transitions refer to the seasonal one-hour clock change. Statistical significance of differences between groups was assessed using the Mann–Whitney U test. Asterisks indicate statistically significant differences (*p < 0.05; **p < 0.01).

Due to the shape of the trend line shown in **Figure 1**, the hypothesis that a transition between standard and daylight saving time may result in changes in the number of hypnotics and sedatives prescribed was also tested. The comparative analysis covered both workdays and all days within two-week intervals preceding and following the clock change (**Figure 2**).

Analyses of daily prescription counts in the two-week periods before (−14 days) and after (+14 days) the daylight saving time change, revealed differences between women and men as well as within subgroups comprising fewer than 259 prescriptions When all calendar days were included, a significant reduction in prescription counts was observed among women following both the summer and winter time changes, b (p < 0.05 for summer and *p* < 0.01 for winter). In contrast, no statistically significant differences were detected among men when all calendar days were analysed. When the analysis was restricted to working days only, among men a significant increase in prescription counts was detected following the winter time change, (p < 0.05). No significant differences were found following the summer time change in men when only working days were considered.

These data suggest transient alterations in the use of hypnotic medication around daylight saving time shifts.

**Figure 3:**
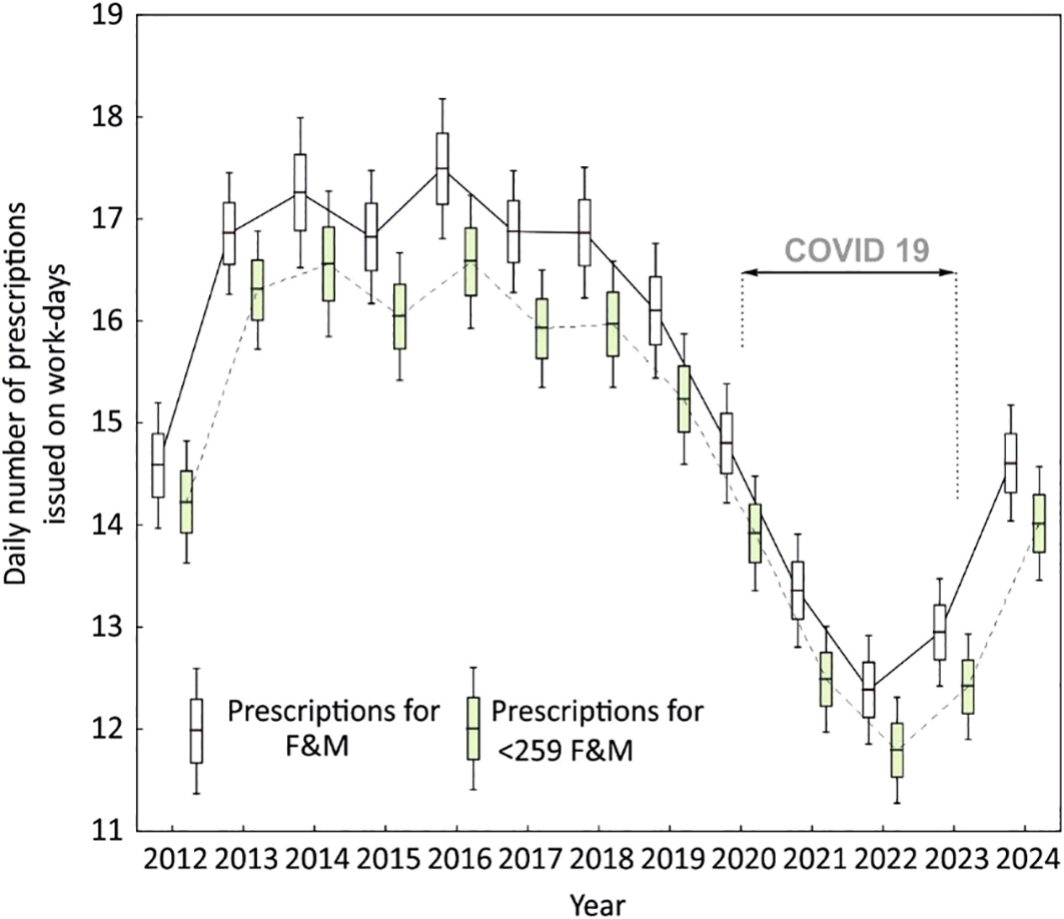
Temporal trends in the daily number of prescriptions issued on workdays between 2012 and 2024. Box-and-whisker plots show annual distributions of prescriptions for all patients (F&M, open boxes) and for subgroups of patients who received fewer than 259 prescriptions (<259 F&M, shaded boxes). Solid and dashed lines connect annual means. A marked decline in prescription counts was observed from 2018 onward, reaching the lowest levels during the COVID-19 pandemic (2020–2022), followed by a gradual post-pandemic increase.

**Figure 4:**
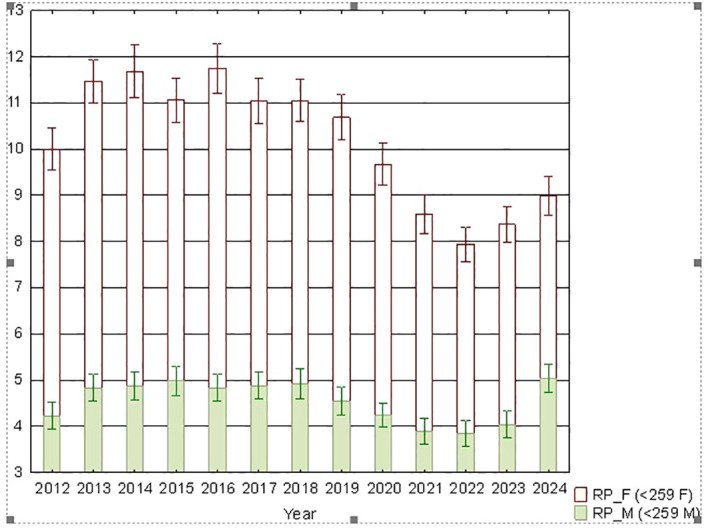
Temporal trends in the daily number of prescriptions issued on working days between 2012 and 2024. Data stratified by gender (F, females; M, males).

As a supplement, **Figure 3** and **Figure 4** present the annual data stratified by gender for the analyzed period (**Figure 4**) and for both genders combined (**Figure 3**).

## Discussion

Our study provides comprehensive data on the prescription of sleep medication in the primary care setting. Data from 2012 to 2024, comprising 3279 workdays and 50 822 prescriptions issued for sleep medication were analyzed.

The collected data reveal a seasonal trend in the number of prescriptions issued for hypnotics and sedatives (**Figure 1**). The number of prescriptions was statistically significantly smaller in summer months (June–August) (**Figure 1**), with the lowest values in July. When analyzing data for all patients (**Table 3**), the statistical difference refers to May-August, The pattern was consistent for both genders yet differed as regards the dynamics and amplitude. In men a decrease in the number of prescriptions begins earlier and is characterized by a greater statistical significance (**Table 4**). This decrease is also deeper, with IRR values of 0.88–0.92, which corresponds to a decrease of 8-12% compared to January. In the female population, a decrease in the number of prescriptions was observed in September, but it was less pronounced, with IRR of 0.94 (Tab. 4). In the female population, there was also a slight but significant increase in the number of prescriptions in February compared to January (IRR 1.06; p = 0.038). The analysis of data from the 2023 meteorological yearbook for the station located Olsztyn, Poland, reveals the most rapid increase in the number of hours of sunshine per day in February (27).

**Table 4:** Incidence rate ratios (IRR) for the effect of calendar month on daily prescription counts in all patients, males, and females (Poisson GLM, workdays 2012–2024).

All patients					Males					Females			
Parameter	IRR	Lower 95% CI	Upper 95% CI	*p*>|z|	IRR	Lower 95% CI	Upper 95% CI	*p*>|z|	IRR		Lower 95% CI	Upper 95% CI	*p*>|z|
Intercept	10.31	10.00	10.63	0.000	3.29	3.11	3.47	0.000	7.02		6.77	7.29	0.000
**February**	1.03	0.99	1.08	0.193	0.97	0.90	1.05	0.451	1.06		1.00	1.11	**0.038**
March	1.02	0.98	1.07	0.310	0.99	0.92	1.07	0.800	1.04		0.99	1.09	0.163
April	1.00	0.96	1.05	0.982	0.94	0.87	1.02	0.150	1.03		0.98	1.08	0.321
**May**	0.96	0.91	1.00	**0.038**	0.92	0.85	0.99	**0.034**	0.97		0.92	1.02	0.284
**June**	0.94	0.90	0.98	**0.006**	0.88	0.81	0.95	**0.001**	0.97		0.92	1.02	0.234
**July**	0.93	0.89	0.97	**0.002**	0.88	0.81	0.95	**0.001**	0.96		0.91	1.01	0.119
**August**	0.94	0.90	0.98	**0.006**	0.93	0.87	1.01	0.086	0.94		0.90	1.00	**0.031**
September	1.00	0.96	1.04	1.000	0.97	0.90	1.05	0.442	1.01		0.96	1.07	0.603
October	1.04	1.00	1.09	0.065	1.02	0.95	1.10	0.588	1.05		1.00	1.11	0.062
November	1.00	0.95	1.04	0.831	0.98	0.91	1.06	0.613	1.00		0.95	1.06	0.932
December	0.99	0.95	1.03	0.628	1.01	0.93	1.08	0.907	0.98		0.93	1.04	0.504

The table presents IRR estimates with 95% confidence intervals and p-values derived from Poisson generalized-linear models with a log link, using January as the reference month. An IRR below 1.0 indicates a lower prescription rate compared with January, while an IRR above 1.0 indicates a higher rate.Bold values indicate statistically significant results (p < 0.05).

When attempting to interpret our results, there is a correlation with the length of the day (shortest in December-January, longest in June-July). According to the existing scientific literature, psychiatric disorders, particularly those associated with anxiety and insomnia, may exhibit significant seasonal variability (7). During the autumn and winter months, an increased prevalence and severity of depressive symptoms are observed (28, 29). In contrast, in the spring period, characterized by increased exposure to sunlight following the winter season, there may be an elevated risk of the onset of bipolar disorder (30). Seasonal fluctuations in symptom severity may also influence prescribing patterns, potentially contributing to increased use of the medications analyzed in the present study.

In mammals, melatonin is produced at night by the pineal gland, and its secretion is inhibited by exposure to light, especially in the wavelength range of 460–480 nm (31). However, in the 21st century, this effect is significantly weakened by ubiquitous artificial lighting and exposure to LED screens. This can result in a delayed sleep onset, which may contribute to the development of sleep disorders and, consequently, may lead to increased prescribing of hypnotics during autumn and winter months. Scientific reports, as well as the described pathomechanism, are, however, ambiguous.

Sivertsen B et all., based on data from northern Norway (Tromsø - 69°39’ N), a region in which differences in daylength are particularly great, reported that in wintertime insomnia was slightly more frequent in men (but not in women) (32). Fridborg O et al. compared disturbances in sleep quality of the Norwegian population (Tromsø) and that of Ghana (the geographical location of the capital, Accra, was used for illustration purposes - 5°56’ N). Insomnia decreased in both analyzed countries from January to August, but was more pronounced in Norway (33). A similar, earlier study of 2001 from this region (Tromsø), suggested that disturbances in sleep quality occur statistically more frequently in the Norwegian population in wintertime (34).

Suzuki M et al, using questionnaires on sleep duration and sleep problems (difficulty initiating sleep [DIS], difficulty maintaining sleep [DMS]/early morning awakening [EMA], and excessive daytime sleepiness [EDS]), also suggested seasonal sleep disorders in the Japanese population (35). This study is interesting since the research areas were located between 40°09’ N (Aomori) and 31°09’N (Miyazaki), that is at latitudes corresponding to European countries in the Mediterranean basin. Their study indicates that seasonal sleep disturbances occur in this population, but they are more pronounced in the summer than in winter. It should be noted that the shortest day in the city of Olsztyn (53°78’ N) in Poland during winter lasts just over 7 hours, while in Aomori over 9 hours, and in Miyazaki nearly 10 hours (9 hours and 53 minutes, to be precise) (36).

The shape and the intersection point of the curves illustrating the daylength and the number of prescriptions issued (**Figure 1**) suggested a link with transitioning between standard and daylight saving time. We verified this hypothesis by analyzing the number of prescriptions in two-week intervals before and after the clock change (**Figure 2**). Transitions between standard and daylight saving time were associated with statistically significant short-term changes in prescription rates. It should be emphasized that the analysis addressed prescribing behaviour rather than a direct assessment of sleep quality, which warrants cautious interpretation of the results.

The observed sex-specific differences further support this interpretation. While a reduction in prescription counts was noted among women following both time changes, a statistically significant increase in prescriptions among men was observed only after the winter time change and exclusively in analyses restricted to working days. This may reflect sex-related differences in symptom reporting, healthcare utilization, or clinical decision-making, particularly during periods of reduced daylight exposure.

Another finding of our study is the decline in the number of prescriptions during the COVID-19 pandemic (**Figure 3** and **4**). During pandemic-related restrictions, patients’ access to medical services was limited, which naturally led to a decrease in the number of consultations and prescriptions issued. We described this phenomenon in 2020, when we assessed the provision of medical services between April and July in the years 2010–2020 (37). In that study, we demonstrated a statistically significant decline in the consultation rate in primary healthcare. Another factor may be the increased number of teleconsultations, which limits the possibility of direct clinical assessment of the patient and, as a consequence, leads to a reduction in the number of prescriptions issued for hypnotic and sedative medications.

Many studies indicated a population-level increase in the prevalence of sleep disorders during the COVID-19 pandemic (38). Moreno CR et all. reported that during the pandemic there was a significant increase in sleep-related problems, and at the same time, only about one-third of study participants sought professional help in this regard (39). However, these studies did not include an analysis of prescriptions for medications with hypnotic properties. The pandemic may have affected medical practice, among other things, by leading to a more cautious approach to prescribing potentially addictive medication. This hypothesis is confirmed by the results of a study published in 2021, in which Ian Nason et al. revealed a statistically significant decrease in the frequency of starting treatment with antidepressants, antipsychotics, and anxiolytics in the early stages of the COVID-19 pandemic (40).

It is quite commonly known that women are more likely to take sleep medication (41). This is also the case in our study (**Figure 4**), and the quantitative relationship exceeding a ratio of 2:1 is also similar (42). An interesting finding, however, is the significant (greater than in men) decline in prescriptions for these drugs among women during the pandemic. This result, like the those of analyses before and after the transitions between daylight saving time and standard time, differs from what was predicted based on reports of sleep disorders and needs to be confirmed in another study (43).

### Limitations of the study

Although our study covers a large amount of data and a long observation period, it has some limitations. It includes data from a single primary healthcare center, which limits the possibility of fully generalizing the results. We analyzed prescriptions that were issued; we do not have information on whether they were actually filled. The lack of detailed clinical information (e.g., comorbidities, duration of symptoms) makes it impossible to assess whether prescriptions were issued for primary or secondary insomnia. Moreover, the analysis does not take into account the adverse effects of medications or the assessment of their clinical efficacy. Additionally, the analysis did not include all medications with hypnotic properties. We selected and included those that, to our knowledge, are most commonly prescribed for the treatment of short-term insomnia in our region (drugs used in individual cases, e.g., phenobarbital, were excluded from the study).

### Practical conclusions and implications

Despite some limitations, the findings of this study provide important insights that are relevant to both clinical practice and health policy making. The analysis covering the years 2012–2024 revealed that the number of prescriptions for hypnotics and sedatives reaches its lowest level in the summer months (May–August) and its highest level in winter and early spring. We also demonstrated that longer days correlate with lower prescription rates of sleep medication. The obtained results suggest a significant impact of transitions between daylight saving time and standard time on the number of prescriptions for sleep medication. As regards gender differences, it was found that in men the decline in the number of prescriptions occurs earlier and is more pronounced (IRR 0.88–0.92), while in women these changes are weaker and only reach statistical significance in August (IRR 0.94). Between 2020 and 2022, covering the COVID-19 pandemic, the number of prescriptions issued for hypnotics and sedatives decreased significantly. This can be interpreted in the context of limited access to healthcare services, the predominance of teleconsultations, and physicians’ more conservative approach to pharmacotherapy during this period.

### Key findings

- Prescriptions for hypnotics and sedatives showed a clear seasonal pattern, reaching their lowest levels in summer and highest in winter and early spring.
- Longer day length was associated with lower prescription rates, suggesting that seasonal light exposure may influence sleep medication use.
- Daylight saving time transitions were associated with sex-specific changes in prescribing patterns, with decreases observed in women after both transitions and a post-winter increase in men (limited to working-day analyses).
- The marked decline in prescription rates during the COVID-19 pandemic may reflect changes in healthcare access and prescribing practices during that period.

## Data Availability

The data analyzed in this study is subject to the following licenses/restrictions: The datasets analyzed in this study contain sensitive health-related information and are subject to data protection and confidentiality regulations. Therefore, they are not publicly available but may be accessed from the corresponding author upon reasonable request, subject to institutional approval. Requests to access these datasets should be directed to MR malgorzata.romaszko.1@gmail.com.

## References

[1] Ahn E Baek Y Park J-E Lee S Jin H-J . Elevated prevalence and treatment of sleep disorders from 2011 to 2020: a nationwide population-based retrospective cohort study in Korea. BMJ Open. (2024) 14:e075809. doi: 10.1136/bmjopen-2023-075809. PMID: 38309764 PMC10840067

[2] Ohayon MM . Epidemiology of insomnia: what we know and what we still need to learn. Sleep Med Rev. (2002) 6:97–111. doi: 10.1053/smrv.2002.0186. PMID: 12531146

[3] Morin CM Benca R . Chronic insomnia. Lancet. (2012) 379:1129–41. doi: 10.1016/s0140-6736(11)60750-2. PMID: 22265700

[4] Auger RR Burgess HJ Emens JS Deriy LV Thomas SM Sharkey KM . Clinical practice guideline for the treatment of intrinsic circadian rhythm sleep-wake disorders: advanced sleep-wake phase disorder (ASWPD), delayed sleep-wake phase disorder (DSWPD), non-24-hour sleep-wake rhythm disorder (N24SWD), and irregular sleep-wake rhythm disorder (ISWRD). An update for 2015: an American Academy of Sleep Medicine clinical practice guideline. J Clin Sleep Med. (2015) 11:1199–236. doi: 10.1007/978-3-030-43803-6_6. PMID: 26414986 PMC4582061

[5] Mattingly SM Grover T Martinez GJ Aledavood T Robles-Granda P Nies K . The effects of seasons and weather on sleep patterns measured through longitudinal multimodal sensing. NPJ Digital Med. (2021) 4:76. doi: 10.1038/s41746-021-00435-2. PMID: 33911176 PMC8080821

[6] Gradiški I Bilić P Sabo T Vilibić M . Tracking insomnia seasonal variations through consumption of hypnotics. Eur Psychiatry. (2017) 41:S283–S. doi: 10.1016/j.eurpsy.2017.02.134

[7] Titova OE Lindberg E Elmståhl S Lind L Benedict C . Seasonal variations in sleep duration and sleep complaints: a Swedish cohort study in middle‐aged and older individuals. J Sleep Res. (2022) 31:e13453. doi: 10.1111/jsr.13453. PMID: 34355440

[8] Sivertsen B Øverland S Krokstad S Mykletun A . Seasonal variations in sleep problems at latitude 63–65 in Norway: the Nord-Trøndelag Health Study, 1995–1997. Am J Epidemiol. (2011) 174:147–53. doi: 10.1093/aje/kwr052. PMID: 21555717

[9] Moynihan R Sanders S Michaleff ZA Scott AM Clark J To EJ . Impact of COVID-19 pandemic on utilisation of healthcare services: a systematic review. BMJ Open. (2021) 11:e045343. doi: 10.1136/bmjopen-2020-045343. PMID: 33727273 PMC7969768

[10] Ftouni R AlJardali B Hamdanieh M Ftouni L Salem N . Challenges of telemedicine during the COVID-19 pandemic: a systematic review. BMC Med Inf Decis Making. (2022) 22:207. doi: 10.1186/s12911-022-01952-0. PMID: 35922817 PMC9351100

[11] Rishi MA Ahmed O Barrantes Perez JH Berneking M Dombrowsky J Flynn-Evans EE . Daylight saving time: an American Academy of Sleep Medicine position statement. J Clin Sleep Med. (2020) 16:1781–4. doi: 10.5664/jcsm.8780. PMID: 32844740 PMC7954020

[12] Rishi MA Cheng JY Strang AR Sexton-Radek K Ganguly G Licis A . Permanent standard time is the optimal choice for health and safety: an American Academy of Sleep Medicine position statement. J Clin Sleep Med. (2024) 20:121–5. doi: 10.5664/jcsm.10898. PMID: 37904574 PMC10758561

[13] Wittmann M Dinich J Merrow M Roenneberg T . Social jetlag: misalignment of biological and social time. Chronobiol Int. (2006) 23:497–509. doi: 10.1080/07420520500545979. PMID: 16687322

[14] Baron KG Reid KJ . Circadian misalignment and health. Int Rev Psychiatry. (2014) 26:139–54. doi: 10.3109/09540261.2014.911149. PMID: 24892891 PMC4677771

[15] He C Anand ST Ebell MH Vena JE Robb SW . Circadian disrupting exposures and breast cancer risk: a meta-analysis. Int Arch Occup Environ Health. (2015) 88:533–47. doi: 10.1007/s00420-014-0986-x. PMID: 25261318

[16] Chen J Peng G Sun B . Alzheimer’s disease and sleep disorders: a bidirectional relationship. Neuroscience. (2024) 557:12–23. doi: 10.1016/j.neuroscience.2024.08.008. PMID: 39137870

[17] Ferguson JM Costello S Neophytou AM Balmes JR Bradshaw PT Cullen MR . Night and rotational work exposure within the last 12 months and risk of incident hypertension. Scandinavian J Work Environ Health. (2018) 45:256. doi: 10.5271/sjweh.3788. PMID: 30614503 PMC6494694

[18] Zhang X Sun Y Ye S Huang Q Zheng R Li Z . Associations between insomnia and cardiovascular diseases: a meta-review and meta-analysis of observational and Mendelian randomization studies. J Clin Sleep Med. (2024) 20:1975–84. doi: 10.5664/jcsm.11326. PMID: 39167428 PMC11609828

[19] Methodology WHOCCfDS . Anatomical Therapeutic Chemical (ATC) Classification System. Oslo, Norway: World Health Organization Collaborating Centre for Drug Statistics Methodology (2025).

[20] Minister Z . Rozporządzenie Ministra Zdrowia z dnia 12 maja 2022 r. w sprawie odwołania na obszarze Rzeczypospolitej Polskiej stanu epidemii. (2022), 1027.

[21] Cameron AC Trivedi PK . Regression analysis of count data. Cambridge, UK: Cambridge University Press (2013).

[22] Hilbe JM . Negative binomial regression. Cambridge, UK: Cambridge University Press (2011).

[23] Von Elm E Altman DG Egger M Pocock SJ Gøtzsche PC Vandenbroucke JP . The Strengthening the Reporting of Observational Studies in Epidemiology (STROBE) statement: guidelines for reporting observational studies. Lancet. (2007) 370:1453–7. doi: 10.1136/bmj.39335.541782.ad. PMID: 18064739

[24] Lang TA Altman DG . Basic statistical reporting for articles published in biomedical journals: the “Statistical Analyses and Methods in the Published Literature” or the SAMPL Guidelines. Int J Nurs Stud. (2015) 52:5–9. doi: 10.18243/eon/2016.9.7.4. PMID: 25441757

[25] Dobson AJ Barnett AG . An introduction to generalized linear models. Boca Raton, FL, USA: Chapman and Hall/CRC (2018).

[26] Seabold S Perktold J . Statsmodels: econometric and statistical modeling with python. SciPy. (2010) 7:92–6. doi: 10.25080/majora-92bf1922-011

[27] Instytut Meteorologii i Gospodarki W . Rocznik meteorologiczny 2023. Warszawa: IMGW-PIB (2023).

[28] Rybakowski J . Forty years of seasonal affective disorder. Psychiatria Polska. (2024) 58:747–59. doi: 10.12740/pp/186721. PMID: 39863986

[29] Diagnostic and statistical manual of mental disorders. Am Psychiatr Assoc. (2013) 21:591–643. Edition F.

[30] Bauer M Glenn T Alda M Aleksandrovich M Andreassen OA Angelopoulos E . Solar insolation in springtime influences age of onset of bipolar I disorder. Acta Psychiatrica Scandinavica. (2017) 136:571–82. doi: 10.1111/acps.12772. PMID: 28722128

[31] Bonmati-Carrion MA Arguelles-Prieto R Martinez-Madrid MJ Reiter R Hardeland R Rol MA . Protecting the melatonin rhythm through circadian healthy light exposure. Int J Mol Sci. (2014) 15:23448–500. doi: 10.3390/ijms151223448. PMID: 25526564 PMC4284776

[32] Sivertsen B Friborg O Pallesen S Vedaa Ø Hopstock LA . Sleep in the land of the midnight sun and polar night: The Tromsø study. Chronobiol Int. (2021) 38:334–42. doi: 10.1080/07420528.2020.1845191. PMID: 33249932

[33] Friborg O Bjorvatn B Amponsah B Pallesen S . Associations between seasonal variations in day length (photoperiod), sleep timing, sleep quality and mood: a comparison between Ghana (5) and Norway (69). J Sleep Res. (2012) 21:176–84. doi: 10.1111/j.1365-2869.2011.00982.x. PMID: 22074234

[34] Husby R Lingjaerde O . Prevalence of reported sleeplessness in northern Norway in relation to sex, age and season. Acta Psychiatrica Scandinavica. (1990) 81:542–7. doi: 10.1111/j.1600-0447.1990.tb05009.x. PMID: 2378246

[35] Suzuki M Taniguchi T Furihata R Yoshita K Arai Y Yoshiike N . Seasonal changes in sleep duration and sleep problems: A prospective study in Japanese community residents. PloS One. (2019) 14:e0215345. doi: 10.1371/journal.pone.0215345. PMID: 30998709 PMC6472875

[36] Time and Date - World Clock, Time Zones, Weather & Astronomy. Stavanger, Norway: Time and Date AS (2025).

[37] Ochal M Romaszko M Glińska-Lewczuk K Gromadziński L Romaszko J . Assessment of the consultation rate with general practitioners in the initial phase of the COVID-19 pandemic. Int J Environ Res Public Health. (2020) 17:7710. doi: 10.3390/ijerph17217710. PMID: 33105612 PMC7659926

[38] Jahrami HA Alhaj OA Humood AM Alenezi AF Fekih-Romdhane F AlRasheed MM . Sleep disturbances during the COVID-19 pandemic: a systematic review, meta-analysis, and meta-regression. Sleep Med Rev. (2022) 62:101591. doi: 10.1016/j.smrv.2022.101591. PMID: 35131664 PMC8782754

[39] de Castro Moreno CR Conway SG Assis M Genta PR Pachito DV Tavares A Jr. . COVID-19 pandemic is associated with increased sleep disturbances and mental health symptoms but not help-seeking: a cross-sectional nation-wide study. Sleep Sci. (2022) 15:1–7. doi: 10.5935/1984-0063.20220027. PMID: 35662970 PMC9153976

[40] Nason I Stein DT Frank RG Stein MB . Decline In New Starts Of Psychotropic Medications During The COVID-19 Pandemic: Study examines new starts of psychotropic medications during the COVID-19 pandemic. Health Affairs. (2021) 40:904–9. doi: 10.1377/hlthaff.2021.00028. PMID: 34097524

[41] Zhang B Wing Y-K . Sex differences in insomnia: a meta-analysis. Sleep. (2006) 29:85–93. doi: 10.1093/sleep/29.1.85. PMID: 16453985

[42] Delpino FM Reis A Minami B Lara N Cechin J . Factors associated with changes in sleep quality and use of sleep medications in beneficiaries of health plans. Sleep Epidemiol. (2023) 3:100056. doi: 10.1016/j.sleepe.2023.100056. PMID: 38826717

[43] Alimoradi Z Gozal D Tsang HW Lin CY Broström A Ohayon MM . Gender‐specific estimates of sleep problems during the COVID‐19 pandemic: systematic review and meta‐analysis. J Sleep Res. (2022) 31:e13432. doi: 10.1111/jsr.13432. PMID: 34245055 PMC8420603

